# A Novel Radiation-Induced p53 Mutation Is Not Implicated in Radiation Resistance via a Dominant-Negative Effect

**DOI:** 10.1371/journal.pone.0087492

**Published:** 2014-02-18

**Authors:** Yunguang Sun, Carey Jeanne Myers, Adam Paul Dicker, Bo Lu

**Affiliations:** 1 Department of Radiation Oncology, Thomas Jefferson University, Philadelphia, Pennsylvania, United States of America; 2 Department of Microbiology and Immunology, Thomas Jefferson University, Philadelphia, Pennsylvania, United States of America; Rush University Medical Center, United States of America

## Abstract

Understanding the mutations that confer radiation resistance is crucial to developing mechanisms to subvert this resistance. Here we describe the creation of a radiation resistant cell line and characterization of a novel p53 mutation. Treatment with 20 Gy radiation was used to induce mutations in the H460 lung cancer cell line; radiation resistance was confirmed by clonogenic assay. Limited sequencing was performed on the resistant cells created and compared to the parent cell line, leading to the identification of a novel mutation (del) at the end of the DNA binding domain of p53. Levels of p53, phospho-p53, p21, total caspase 3 and cleaved caspase 3 in radiation resistant cells and the radiation susceptible (parent) line were compared, all of which were found to be similar. These patterns held true after analysis of p53 overexpression in H460 cells; however, H1299 cells transfected with mutant p53 did not express p21, whereas those given WT p53 produced a significant amount, as expected. A luciferase assay demonstrated the inability of mutant p53 to bind its consensus elements. An MTS assay using H460 and H1299 cells transfected with WT or mutant p53 showed that the novel mutation did not improve cell survival. In summary, functional characterization of a radiation-induced p53 mutation in the H460 lung cancer cell line does not implicate it in the development of radiation resistance.

## Introduction

Lung cancer is the number one cause of cancer-related deaths in the United States, and account for 2.4% of all deaths worldwide [1]. In 2009, over 200,000 new cases of lung cancer were diagnosed, the majority of which were non-small cell lung cancer (NSCLC) [2]. 2/3 of these patients will be given radiotherapy (RT) as part of their treatment regimen. The success of RT in lung cancer is greatly affected by a variety of factors including location, size, grade, extent of invasion, and individual tumor characteristics. Unfortunately, some of the cellular damage that causes cancer can also induce resistance to treatments, making it a very real concern. Understanding the genetic origins of these mutations is crucial to developing mechanisms to subvert this resistance.

With the advent of genomic sequencing techniques, identifying mutations in tumor samples has become commonplace, but this influx of information does not always clearly indicate which of the anomalies identified, if any, is responsible for therapeutic resistance. Thus, many labs have begun engineering cell lines expressing single mutations and using these to examine chemo- and radioresistance. These lines can be studied directly. Alternatively, some groups use molecular modeling to predict which mutations will have functional consequences.

One of the most studied genes involved in cell cycle control is p53, which operates as a cell cycle monitor; it has been implicated in the regulation of both the G1/S and G2/M checkpoints via p21 [3]. p53 can also induce the caspase cascade, resulting in the cleavage of caspase 3 and apoptosis [4]–[7]. Mutations in p53 have been implicated in the development of ∼50% of cancers, including breast, colon, skin, brain, stomach, cervical, liver esophageal, bladder, and lung cancers [8]–[32]. Studying these mutations has revealed that many are also involved in radiation- and chemoresistance.

We hypothesize that radiation resistance results from genetic changes in cancer genomes, and have established a radiation-resistant NSCLC cell line to serve as a cell model for us to understand the molecular mechanism. Ion torrent analyses on this cell line, in comparison with its original clone, identified several mutations. One of these is a novel deletion mutant in p53. We then performed functional characterization of this mutation to determine its role in resistance to radiotherapy, and determined that it was not a mechanism of resistance when WT p53 is also present.

## Results

### Discovery and characterization of a novel radiation-induced p53 deletion

**Figure 1:**
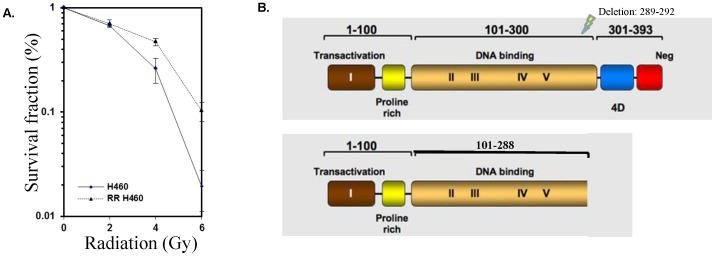
Identification of a novel radiation-induced p53 mutation that confers resistance to subsequent radiotherapy. H460 cells (p53-competent) were exposed to sequentially higher doses of radiation, then incubated at 37°C for 8–10 days. One surviving (radiation-resistant) clone was selected and expanded to create a radiation-resistance H460 cell line (RR-H460). To confirm the effect of novel deletion on cell survival, a second radiation of 6 Gy was applied to cells from the parental and RR lines. A. Radiation resistance was confirmed by clonogenic assay after treatment with 0–6 Gy. A significant (p<0.007) difference in survival between the radiation resistant colony and the parent cell line was noted at doses of 4 or 6 Gy. SF = 0.2. B. Sequencing analysis revealed a novel deletion mutation at the end of the DNA binding domain of p53. Figure modified from the TP53 website (p53.free.fr).

**Figure 2:**
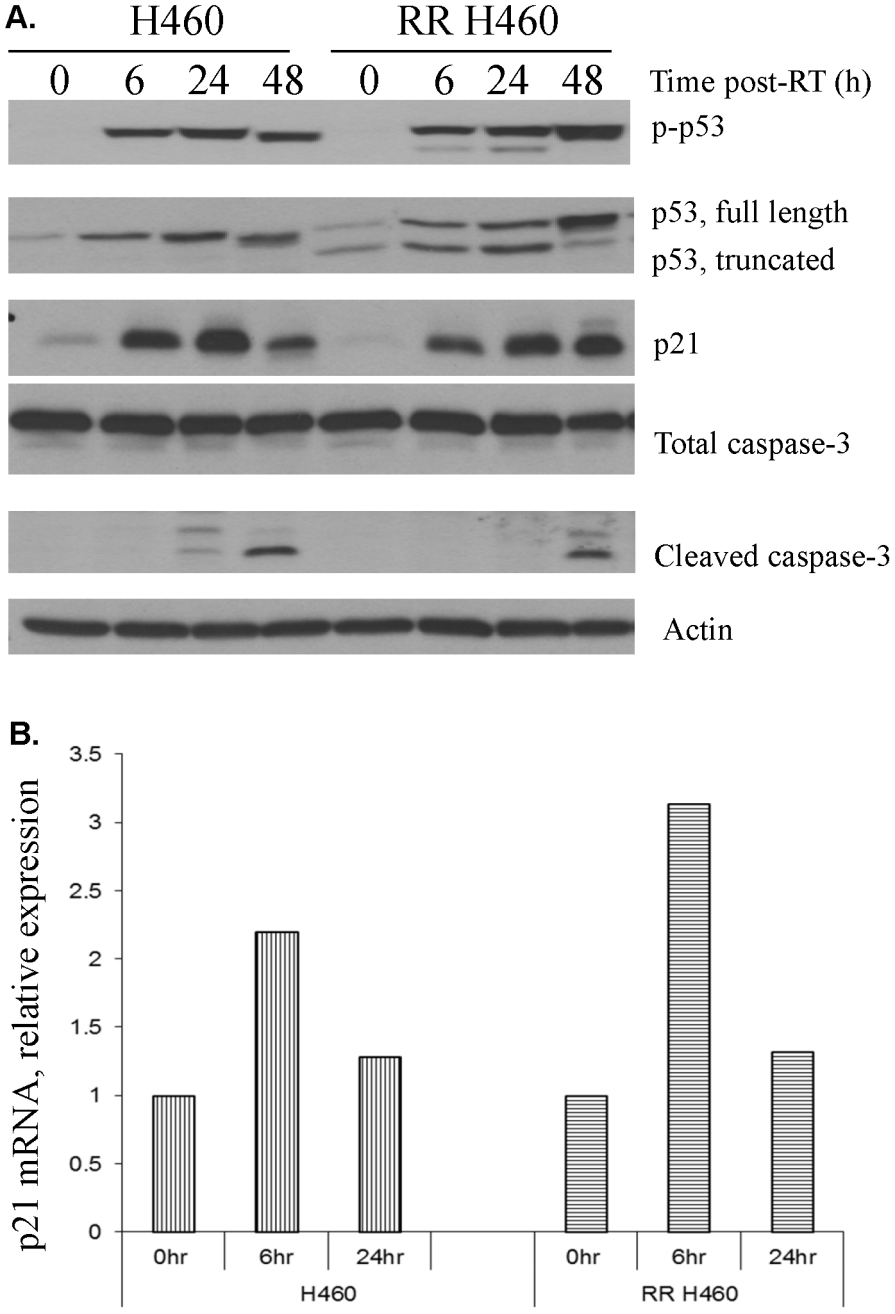
Characterization of parent and RR cell line after treatment with 6 Gy radiation A. Subsequent protein expression was analyzed by Western blot. The parent and derivative cell lines were found to have comparable levels of total caspase 3. Expression of p53, phosphorylated p53, p21, and caspase cleavage were all delayed in the radiation resistant cell line. The radiation resistant cell line clearly shows two forms of p53, mutant and WT. B. mRNA analysis of p21 expression indicates that, in the hours immediately after irradiation, transcription is initially higher in the RR cell line, but by 24-irradiation the levels are similar in the RR and parent cell lines.

The H460 lung cancer cell line has intact p53; treatment with 20 Gy radiation produced a small number of radiation-resistant (RR) surviving cells, which were collected for study. Radiation resistance was confirmed by clonogenic assay (Fig. 1A), showing a significant difference (p<0.007) in survival between the radiation resistant cells and the parent cell line at doses as small as 4 Gy. Sequencing analysis revealed a novel deletion at the end of the DNA binding domain of p53 (Fig. 1B). This 4 amino acid deletion generates a stop codon, resulting in a truncated protein missing the C-terminal ∼100 amino acids. The presence of the p53 deletion was confirmed by Sanger sequencing, and the corresponding p53 deletion mutant protein was identified and confirmed by Western blot analysis (Fig. 2).

### Characterization of a novel p53 deletion mutant

To determine the effect of our novel deletion on cell survival and the expression of p53 and downstream effector molecules responsible for cell cycle arrest and apoptosis, a second radiation of 6 Gy was applied to cells from the parental and RR cells. Subsequent protein expression was analyzed by Western blot (Fig. 2). The parent and derivative cell lines were found to have comparable levels of p21, total caspase 3 and cleaved caspase 3. Levels of p53 and phosphorylated p53 were also comparable. As expected, the radiation resistant samples showed two bands, corresponding to normal and mutant p53 and their phosphorylated counterparts.

Notably, the expression of both p21 and cleaved caspase 3 is delayed in the RR cell line. In the parent cell line, cleaved caspase 3 is apparent within 24 h of irradiation; in the RR-H460 line, cleavage is delayed until 48 h post-irradiation. mRNA analysis of p21 expression indicates that, 6 h post-irradiation, transcription is higher in the RR cell line, but by 24 h post-irradiation the levels are similar in the RR and parent cell lines (Fig. 2B).

### p21 does not undergo significant transcriptional activation by the deletion mutant

**Figure 3:**
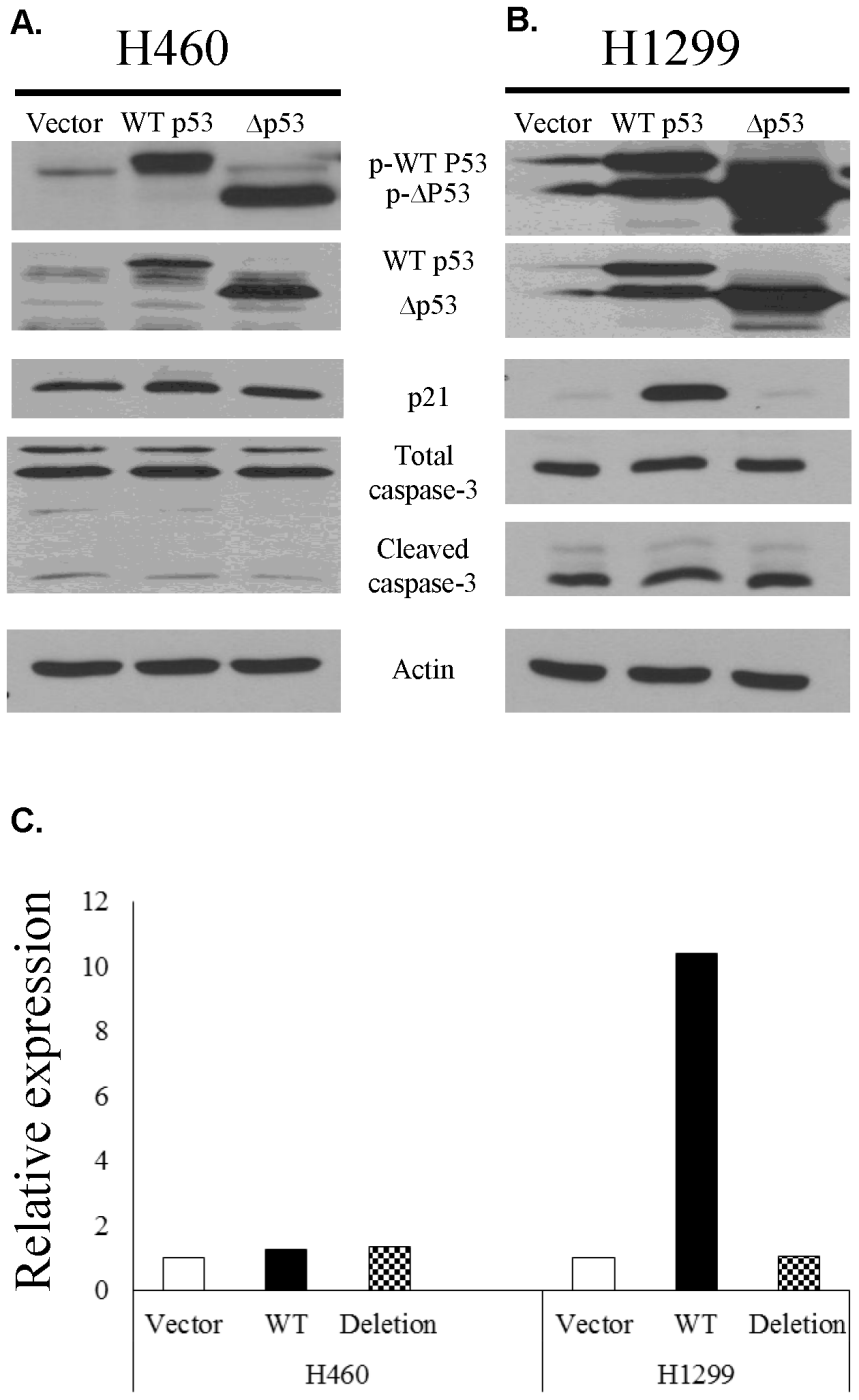
Overexpression of the novel mutant p53 does not induce p21 expression in p53-deficient H1299 cells. A. Overexpression of WT p53 or the deletion mutant in H460 cells resulted in higher expression of the truncated protein than the full length transcript; comparable levels of phosphorylated p53, p21, total caspase 3 and cleaved caspase 3 were observed. B. Overexpression of the same in H1299 cells resulted in similar protein expression patterns with the exception of p21, which was expressed as a result of transfection with WT p53 but not the deletion mutant. C. RT-PCR analysis confirms that similar levels of p21 are present in H460 cells transfected with the WT or mutated p53 and that H1299 cells transfected with WT p53 express much higher levels of p21 than those transfected with the mutant.

When WT or mutant p53 was overexpressed in the parental H460 cell line, comparable levels of phosphorylated p53, total caspase 3 and cleaved caspase 3 were expressed (Fig. 3A); higher amounts of the truncated protein were produced as compared to the full length transcript. A very minor increase in p21 expression was also noted. Transfection of H1299 cells, which are naturally p53 deficient, with mutant overexpressing plasmid did not induce p21 expression, whereas those given WT p53 produced a significant amount (Fig. 3B). Total and cleaved caspase 3 levels were comparable. RT-PCR analysis confirmed that similar levels of p21 were present in H460 cells transfected with the WT or mutated p53 and that H1299 cells transfected with WT p53 expressed much higher levels of p21 than those transfected with the mutant (Fig. 3C).

### Deletion mutant does not impact cell survival unless expression in isolation

**Figure 4:**
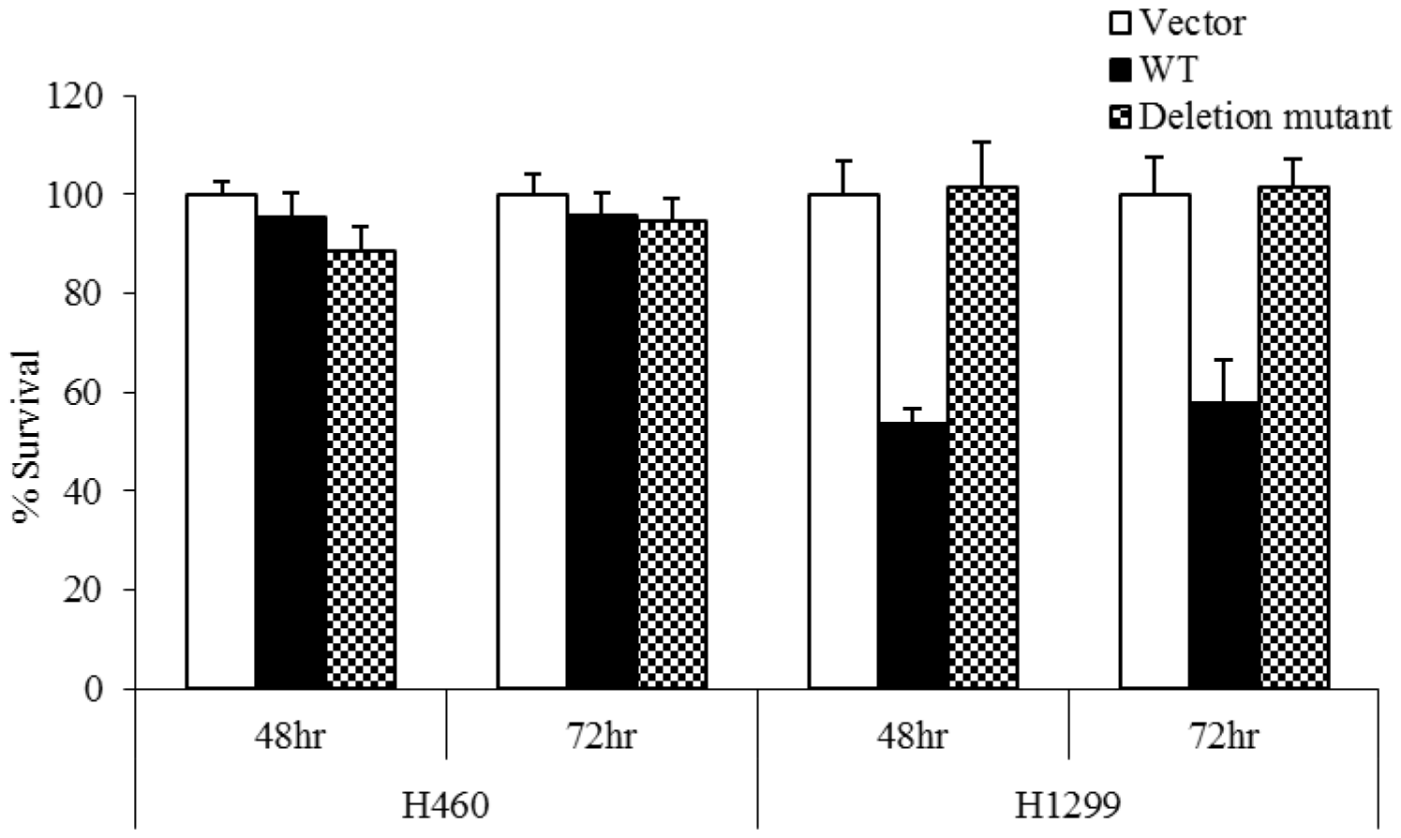
Deletion mutant fails to improve cell survival when expressed in conjunction with WT p53. 48%, whereas transfection with mutant p53 resulted in over 90% survival. In H460 cells, survival was over 90% regardless of p53 mutation status, showing definitively that the novel mutation did not improve cell survival unless present as the only available form of p53, and confirming its inability to act as a dominant negative mutation.

The effect of this novel deletion on cell proliferation was analyzed using an MTS assay. 48 h or 72 h after transfection with WT p53, the survival rate for H1299 cells was 50%, whereas transfection with mutant p53 resulted in over 90% survival (Fig. 4). In H460 cells, survival was over 90% regardless of p53 status, showing definitively that the novel deletion did not improve cell survival when expressed in conjunction with WT p53, and did not act as a dominant negative in the presence of WT p53.

### Overexpression of deletion mutant does not confer radiation resistance

**Figure 5:**
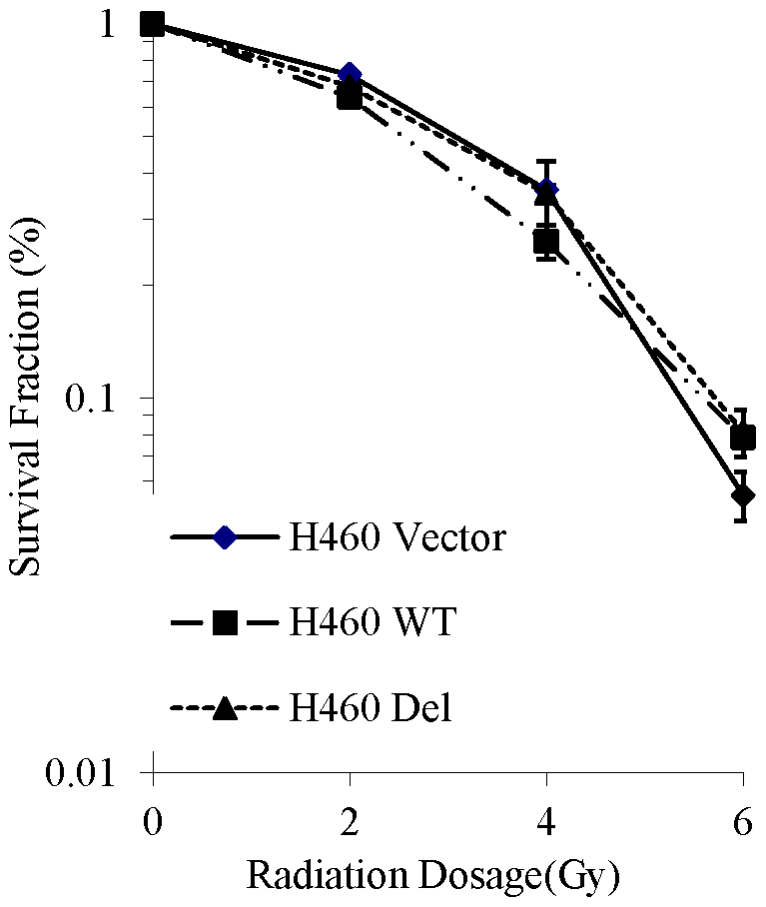
Overexpression of novel p53 mutation does not confer radiation resistance. p53 variant does not affect radiation resistance in the presence of WT p53. Transfection with WT p53 results in slightly decreased survival after irradiation with 4: (SF = 0.2); WT: 1.05 (p = 0.074); DEL: 0.97 (p = 0.27).

Transfection of p53-competent cells with mutant p53 does not change the survival capabilities of H460 cells after low-dose radiation (Fig. 5).

### Mutant p53 does not bind consensus elements

**Figure 6:**
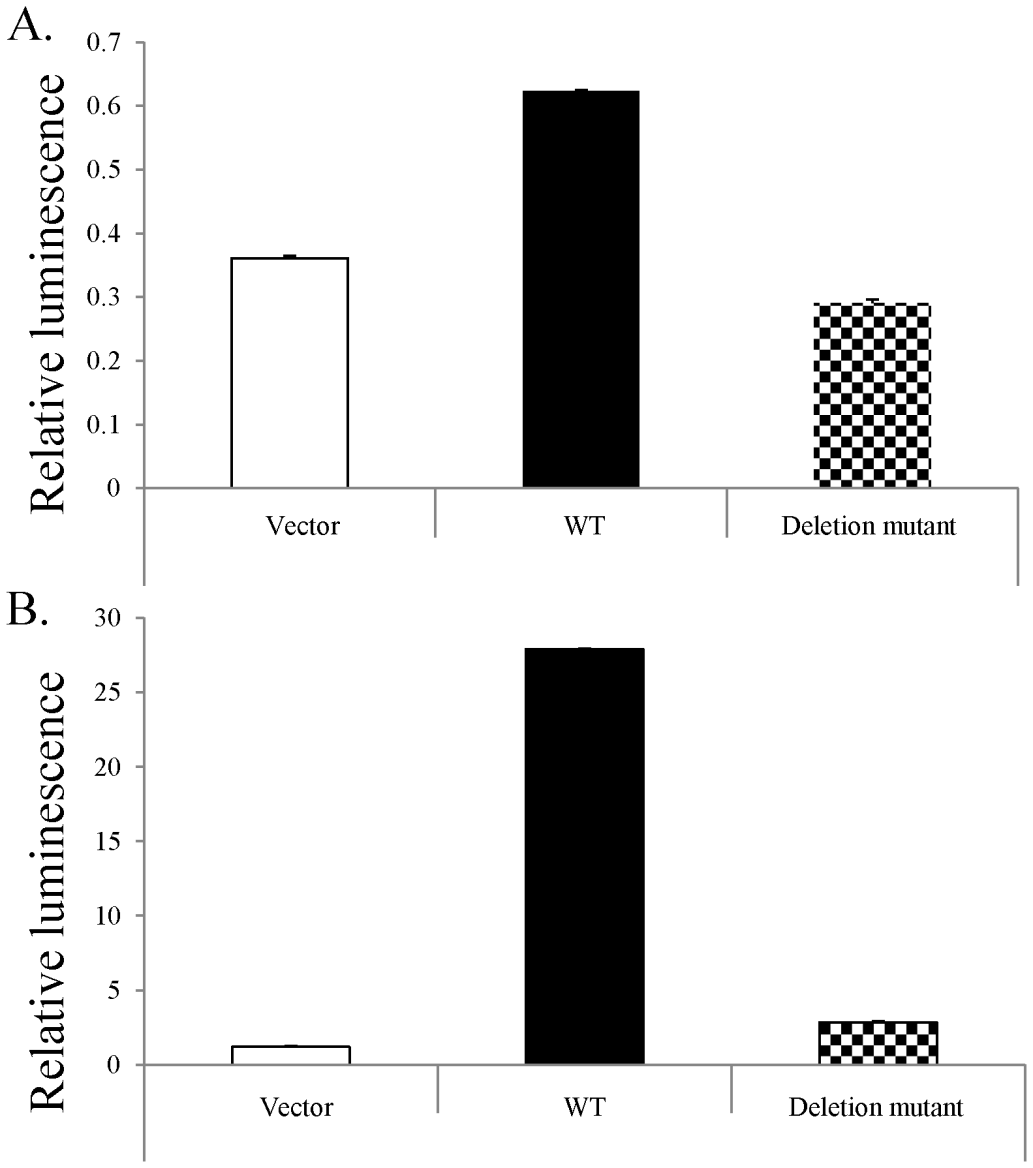
Novel mutation abrogates p53 binding. Cells were transfected with a plasmid containing either WT p53 or that containing the novel mutation. A. After transfection into p53 competent H460 cells, a luciferase assay shows WT p53 is capable of binding its consensus elements, whereas mutant p53 is not. B. When the same vectors were applied to naturally p53-deficient H1299 cells, this difference was much more pronounced. The sample transfected with the mutant p53 showed a small amount of luciferase activity, approximately 3× that of the vector, whereas the WT p53 induced 25× as much.

The development of luciferase reporter vectors has made analysis of the effects of mutation on the binding ability of various transcription factors quick and quantifiable. Examination of H460 cells transfected with WT p53 showed nearly twice the luciferase expression as the control, whereas cells transfected with the mutant p53 had slightly lower expression than the control (Fig. 6). When the same vectors were applied to naturally p53-deficient H1299 cells, the sample treated with the mutant p53 showed a small amount of luciferase activity, approximately 3× that of the vector, whereas the WT p53 induced 25× as much. These luciferase assays demonstrated the inability of mutant p53 to effectively bind its consensus elements, as evidenced by the failure to initiate downstream gene transcription.

## Discussion

This paper details the development of a radiation-resistant NSCLC cell line, the identification of a novel p53 deletion mutant, and subsequent functional characterization of this mutant. Ultimately, while this novel mutant does have interesting subcellular effects, it does not confer any survival advantage to cancer cells when the cells also express WT p53.

Loss of the ability to control cell proliferation is the defining characteristic of cancer; the proteins involved in this process are thus critical to regulating growth and maintaining appropriate proliferative capabilities. In 1979, one of the key proteins involved in this process was discovered: p53 [11]–[13], [30], [33]. In its role as a transcription factor, p53 has been shown to interact with over 100 other proteins, including Nfκb, which is involved in transcription; BRCA1 and PARP 1, which are responsible for DNA repair; and Mdm2, which directly suppresses p53 activity [34]–[45]. Wild-type p53 has a multitude of functions, all of which are involved in regulating the cell cycle and suppressing neoplastic growth, and which can be activated by a variety of pathways triggered by cellular stress, including DNA damage and hypoxia [15], [46]. These functions include initiating cell cycle arrest and DNA repair, which can inhibit the growth of potentially tumorigenic cells by moving them to a state of senescence and/or apoptosis [47]–[48]. As such, the integrity of p53 can determine the function or dysfunction of hundreds of downstream effectors [46]. Thus, when we discovered a novel p53 mutation in a radiation-resistant cell line, we chose to focus our analysis on how it would affect the survival of lung cancer cells.

Approximately 50% of all cancers have some type of mutation in p53. ∼75% of these mutations are missense mutations that affect DNA binding, thus disabling the transcriptional function of p53 [15], [22], [32]. The rest disrupt protein folding, effectively destabilizing it [22]. The International Agency for Research on Cancer currently has over 25,000 reported p53 mutations in their database [22]. While most of these mutations are disabling, some may lead to new functions for p53 that actually enhance tumorigenic growth via aberrant DNA or protein binding [22], [32]; this may also enhance a tumor's invasive capabilities [46]. There have also been reports of p53 mutations that result in EGRF inhibitor resistance [3]. Predominant are single base pair alterations [19]; in lung cancer, these are most commonly G→T transversions [10], [19]. The variety of mutations that have been described indicates that different subtypes of cancer may have different biochemical origins [14].

p53 inactivation is typically the result of small changes in sequence that result in dominant negative mutant forms of the protein (90%) [49]. Such mutations have the potential to affect a developing cancer in 2 ways: increasing DNA repair processes and thus stunting tumor growth, or, by functional loss, allow the cell to become more permissive regarding DNA damage. There are over 100 potential sites of damage known to cause phenotypic mutations. As the whole protein consists of only 393 amino acids, this indicates that the majority of mutations will result in some type of altered function. Statistically, most p53 mutations occur at positions 158, 175, 248 and 273 [50]–[51]; the mutation reported herein, a deletion of bases 298–292, has never before been reported in the literature. This deletion, located at the end of the DNA binding domain of p53, generates a stop codon, resulting in a truncated protein missing the C-terminal ∼100 amino acids, which includes the tetramerization domain (TD) and the C terminal regulatory domain. Comparatively, mutations in these domains are rare compared to those in the DNA binding domain [18]–[19], [23], [28]. p53 in its active form preferentially acts as a tetramer; this domain is facilitates appropriate post-translational modification, proper DNA binding, and even degradation. Chene notes that even a single mutation in the TD can have effects as severe as those in the DNA binding domain [52]. Effects on post-translational modification usually result in the failure to phosphorylate [53]–[54], or the inability to ubiquinate appropriately [44], [55]. However, the TD is not absolutely required for DNA binding [56]–[62], due to the cooperative binding of monomers (though this results in up to a 100-fold decrease in affinity [56]. When TD mutations are found in cancer, they are usually (22%) found at the Arg342 residue and result in truncated proteins [18].

Mutations in p53 have been discovered in nearly every type of cancer [8]–[32]; surprisingly, only a small number of these mutations are in the DNA binding domain (see Table 1). While many of these mutations appear during the development of a primary cancer, radiation-induced p53 mutations have also been implicated in a variety of secondary malignancies [1], [25], [63]–[74]. The H460 large cell lung cancer cell line is a hypotriploid human cell line (modal number = 57) with normal p53 expression. Previous publications have indicated that p53 status in H460 cells affects survival post-radiation; Jung et al concluded that increased radiosensitivity was mediated by PTEN expression via p53, and Lee et al found that p53-inducible protein 3 was downregulated in radiation-resistant H460 cells and its overexpression had significant effects on radiosensitivity [75]–[76]. Thus, when we generated a radiation-resistant H460 cell line and discovered a novel p53 mutation, its role in this resistance was of great interest.

**Table 1:** p53 mutations in cancer.

	Number of p53 mutations with reported effects	Number of nonsense mutations (% of total)	Number of mutations in DNA binding domain (% of total)
**All cancers**	28581	2326 (8.14)	567 (2.06)
**Lung cancers**	3075	277 (9.01)	58 (1.94)
**Lung AC**	819	60 (7.33)	19 (2.42)
**Lung SCC**	942	100 (10.62)	16 (1.76)
**M lung AC**	22	0(0)	2 (100)
**M lung SCC**	1	0	0

AC: adenocarcinoma.

SCC: squamous cell carcinoma.

After exposure of H460 cells to 20 Gy of radiation (less than the typical total therapeutic dose; [77], we collected the surviving cells for analysis, and confirmed resistance to subsequent lower doses of radiation (Figure 1A). Sequence analysis of the selected clone revealed a unique p53 mutation resulting in a truncated protein. This nonsense mutation results in the loss the C-terminal ∼100 amino acids, a sequence encoding the oligomerization domain and the nuclear localization signal within it (see Figure 1B).

Comparison of the parent and radiation resistant cell lines after treatment with 6 Gy radiation confirmed the presence of two forms of p53 in the resistant cells (Figure 2A). Total caspase 3 production is comparable in both lines, but cleavage is delayed in the radiation resistant line; p21 expression is also slightly delayed. Interestingly, the level of p21 mRNA is higher in the radiation resistant line in the hours immediately after irradiation (Figure 2B), but at 24 h the two lines have approximately equal expression. As this is not reflected in the level of protein expression, there must be differential regulatory factors between the two lines. This could be expressed either as a difference in gene transcription or in the stability of the RNA itself.

In order to eliminate the effects of innate p53 expression, we constructed an adenovirus-based vector containing either WT p53 or the sequence of the novel mutated form. Transfection with these viral constructs allows overexpression of WT or mutated p53, after which we analyzed the production of downstream proteins p21 and caspase 3 (Figure 3A and B). Transfection of p53-competent cells with the WT adenovirus (Fig. 3A) does not show an increase in expression of p53 itself. The innate ability of H460 cells to produce WT p53, and thus p21, implies that there is also a functioning regulatory system in place to modulate the levels of these proteins being produced. This result is probably due to the innate feedback mechanisms present in these cells that are lacking in H1299 (p53-deficient) cells.

Unlike in the H460 system, p21 expression in H1299 cells was only faintly detectable after transfection with the p53 mutant, but strongly expressed after WT p53 was introduced (Fig. 3A and B); this pattern was confirmed via RT-PCR (Fig. 3C). However, the downstream target, caspase 3, is still produced in similar amounts to that induced by WT p53, indicating that another, p53-independent, process contributes to the induction protein expression in this cell line. Interestingly, though the level of total caspase 3 is similar in both the p53 competent and deficient cell lines, H1299 cells show much greater levels of cleaved caspase 3. This can be attributed to a higher innate sensitivity to transfection toxicity.

The ultimate test of a novel mutation implicated in the development of cancer is to examine the effect on cell proliferation. To address this, we assessed the effect of overexpression of the novel mutant on the survival of p53-competent or –deficient lung cancer cells. Transfection of naturally p53-deficient H1299 cells with WT p53 decreases survival by 40%; this effect was not seen in p53-competent cells (Fig. 4). H460 cells had over 90% survival regardless of the type of p53 transfected, indicating that long term survival is not significantly affected by the novel mutant in the presence of WT p53; this confirms that this novel deletion indeed does not function as a dominant negative. However, it also does not confer any survival advantage. When these transfected cells were exposed to increasing doses of RT, no appreciable difference was seen in H460 cell survival (Fig. 5). At lower radiation doses, WT p53 conferred a slightly significant survival advantage in H1299 cells; however, after a dose of 6 Gy, there was no difference in the survival of H1299 cells, regardless of p53 mutation status.

Given the location of the reported deletion, we expect that there might be two potential effects: abrogation of oligomerization and of DNA binding.

The oligomerization domain is required for maximal p53 function, as it is most physiologically active when condensed into tetramers [78]. Assessment of oligomerization efficiency can be estimated by quantifying downstream protein expression. Normal p53 tetramers function in cell cycle regulation via the induction of p21 and the caspase cascade. Given this understanding regarding the requirements for normal function, it is not surprising that the mutant cell line being studied here showed delayed p21 and caspase 3 expression (Fig. 2A). By the time p21 production has reached its peak in the radiation resistant cells, it is already waning in cells from the parent line; similarly, cleaved caspase 3 is detectable within 24 h in the parent line, but not before 48 h in the radiation resistant cells. Presumably, this novel deletion is eventually compensated for by the persistence of one normal copy of the p53 gene, indicating that a loss of homozygosity delays the expression of apoptotic proteins, but ultimately is insufficient to confer RR. This is surprising in light of the fact that the majority of reported p53 mutations exhibit a dominant negative phenotype [8], [79], which provides a mechanism for their ability to control cell cycle events even in a heterozygous state. Figure 2A confirms the presence of residual normal p53 in the radiation resistant (RR) cell line and the eventual production of similar amounts of p21 and cleaved caspase 3. At 24 h, there is evidence of more apoptosis (ie cleaved caspase 3) in the parental cell line, but total caspase expression is equivalent. Thus, survival difference (see Fig. 1A) is not due to apoptosis. Interestingly, p21 mRNA is actually greater in the RR cells soon after irradiation (Fig. 2B). The lag in expression must be due to other factors, one of which may be irradiation itself [12], [80].

The ability of p53 to bind its consensus elements is critical to its function; without this binding, there can be no signaling induction. In order to assess the effect of our novel deletion on p53 binding, we used a luciferase assay to quantify the ability of cells transfected with either WT or mutant p53 to instigate protein expression. In the pGL3 luciferase reporter system (Promega), the gene of interest is inserted into a vector containing a luciferase gene downstream of the binding elements, providing a simple way to quantify the induction of transcription post-consensus element binding. In order to eliminate the effects of innate p53 expression, as in the H460 cell line, we overexpressed either WT or mutated p53 in H1299 cells, non-small cell lung carcinoma cells with homozygous p53 dysfunction (ATCC), via modified adenovirus. Transfection of H460 and H1299 cells with a modified adenovirus containing either WT or mutated p53indicated that, as expected, this novel deletion decreases the ability of p53 to bind DNA. When (WT) H460 cells' p53 content was boosted by transfection with WT p53, luciferase expression doubled (Fig. 6). Adding mutant p53 actually resulted in slightly reduced luminescence as compared to the vector control, which is consistent with previous observations that most p53 mutations are dominant negative [81]–[85], but not with our data indicating that WT p53 is eventually able to compensate for this deletion. This difference can be resolved by previous observations that, while mutations in the DNA binding domain can exhibit the dominant negative effect [8], [86]–[87], this is lost when the oligomerization domain is mutated [88]–[89]. Presumably, p53's dominant negative status depends on its ability to combine with WT p53 via this domain. p53-deficient H1299 cells showed a small increase in luciferase activity after the addition of mutant p53, significantly less than that induced by transfection with WT p53 (Fig. 6). Taken together, this data indicate that mutant p53 is not able to efficiently bind its consensus elements.

In summary, we have generated a radiation resistant cell line with a unique mutation resulting in a truncated p53 protein with an altered DNA binding domain and deleted oligomerization domain. This deletion causes a loss of function in the resulting protein, as evidenced by the inability to induce the downstream target p21, but also prevents the mutant protein from binding with residual normal p53, thus abrogating its ability to act as a dominant negative. Functional characterization of this novel radiation-induced p53 deletion in the p53-competent H460 lung cancer cell line does not implicate it in the development of radiation resistance in the presence of a WT p53, as it ultimately does not affect cell survival in a heterozygous mutant. During subsequent, more extensive sequencing, additional mutations were identified in the cellular genome of the radiation resistant colony; one of these other genomic alterations may be responsible for the radiation resistance seen in these cells. As the loss of p53 activity characterizes its role in the development of cancer, and its ability to override normal p53 functions can have serious consequences, the identification of mutants that do not act as dominant negatives may have important effects on treatment regimens for cancer patients.

## Materials and Methods

### Ethics statement

None of the experiments in this paper require the approval of an ethics committee; no patient samples or animals were used.

### Cell culture and reagents

The human NSCLC cell line NCI-H460 (H460) and NCI-1299 (H1299) were obtained from the American Type Culture Collection (ATCC). The cells were cultured in an environment of 5% CO_2_ at 37°C in RPMI 1640 (Invitrogen) supplemented with 10% fetal bovine serum.

### Creation of a radiation-resistant cell line

Radiation resistant H460 cells (RR-H460 cells) were selected. Briefly, after H460 cells were treated with 20 Gy using a PanTak 310-keV X-ray machine, cells were cultured for seven days; the surviving cells were trypsinized and cultured in 0.8% methylcellulose supplemented with 20 ng/mL EGF (BD Biosciences), βFGF, and 4 µg/mL insulin (Sigma). EGF, βFGF (20 ng/mL), and insulin (4 µg/mL) were added every second day for 14 days to allow the cells to form spheres. Spheres were diluted with PBS to make a single-cell suspension and then plated in 100 mm dishes with RPMI 1640 supplemented with 10% FBS. A single plaque was chosen for expansion and subsequent characterization.

### Ion torrent sequencing

Limited sequencing was performed on the resistant cell line created and compared to the parent cell line. A sample from the expanded clone was run on an Ion 314 chip in an Ion Torrent PGM System.

### Clonogenic survival assay

Radiation resistance was confirmed by clonogenic assay. Cells were irradiated with 0–6 Gy (dose rate of 1.8 Gy/min) using ^137^Cs irradiator (J.L. Shepherd and Associates). After irradiation, cells were incubated at 37°C for 8–10 days. Cells were fixed for 15 m with 3∶1 methanol/acetic acid and stained for 15 m with 0.5% crystal violet (Sigma) in methanol. After staining, colonies were counted by the naked eye (cut-off of 50 viable cells). The surviving fraction was calculated as (mean colony counts)/(cells inoculated)×(plating efficiency), with plating efficiency defined as (mean colony counts)/(cells inoculated for irradiated controls). The dose enhancement ratio (DER) was calculated as the dose (Gy) of radiation that yielded a surviving fraction of 0.2 for H460 cells divided by that for RR-H460 cells.

### Cell viability assay

An MTS assay was performed using tetrazolium based CellTiter 96® AQueous One Solution Cell Proliferation assay (Promega). H460and H1299 cells were seeded in 96 well plates at 3,000cells/well. Cells were transfected with various plasmids and MTS assay was performed at the indicated times.

### Western blot analysis

Cells were washed twice with ice-cold PBS and then lysed in M-Per mammalian lysis buffer (Thermo Scientific). The protein concentration of the lysates was determined with the Bradford reagent (Bio Rad), and equal amounts of protein were subjected to SDS-PAGE of a 10% or 15% gel. Separated proteins were transferred to a nitrocellulose membrane, which was then exposed to 5% nonfat dried milk in TBS containing 0.1% Tween 20 (0.1% TBST) for 1 h at room temperature and incubated overnight at 4°C with antibodies against caspase-3, phosphor-p53 (Cell Signaling Technology), total p53, p21 (Santa Cruz Biotechnology) or actin (Sigma). The membranes were then washed with 0.1%TBST before incubation with horseradish peroxidase–conjugated goat antibodies to rabbit or mouse (Santa Cruz Biotechnology). Immune complexes were detected with chemiluminescence reagents (Perkin-Elmer Life Science).

### RNA extraction and RT-PCR

Cells were plated in 6-well plates and allowed to reach 80% confluency. 1 mL of Trizol (Invitrogen; Carlsbad, CA) was added into each well, and then RNA was extracted following the manufacturer's guidelines. RNA was further purified by the RNAeasy kit (Qiagen). 2 µg of total RNA was reverse transcribed using hexamer primer and Superscript III reverse transcriptase (Invitrogen, Carlsbad, CA, USA) in a final volume of 20 µl. 2 µl of the cDNA was used for the PCR reactions to amplify the p21 gene with ABI 7500 fast Real-Time System (Invitrogen). The sequences of primers used were described previously [90]. Actin was used as a control.

### Plasmids, site-directed mutagenesis, transfection and dual-luciferase assay

p53 expression plasmid was kindly provided by Dr. Steven McMahon at Thomas Jefferson University. p53 deletion plasmid was made using QuikChange II Site-Directed Mutagenesis Kits (Agilent Technologies, Inc. Santa Clara, CA) to delete nucleotides from 866 to 876. The primer sequences for deletion are as follows:

Forward Primer: 5′-GCACAGAGGAAGAGAATCGGGGAGCCTCAC-3′

Reverse Primer: 5′-GTGAGGCTCCCCGATTCTCTTCCTCTGTGC-3′.

H460, RR-H460 and H1299 cells were transfected 24 hr after seeded in 6-well plate. Plasmids (1.5 µg) in 100 µl of serum-free, antibiotic-free, opt-MEM (Invitrogen) were mixed with 5 µl Lipofectamine 2000 transfection reagent (Invitrogen) dissolved in 100 µl of the same medium and allowed to stand at room temperature for 20 min. The resulting 200 µl transfection solutions were added to each well containing 2 ml medium. Six hours later, the cultures were replaced with 2 ml fresh medium supplemented with 10% FBS and antibiotics. For western blot, cells were collected after and additional 48 hr. PG13-luc (wt p53 binding sites) was purchased from Addgene (Cambridge MA), wt p53 binding sites were removed by digesting with Apa I and Xho I to make pg13-Luc as a non-promoter control. H460 and H1299 cells were plated at 2×104/well in 24-well format. 200 ng total DNA were transfected with 0.6 µl lipofectamine 2000 (Invitrogen) and Renilla Tk (Promega, Madison, WI) was used as a transfection efficiency control. Luciferase activity is detected by dual-luciferase kit (Promega).

### Statistical Analysis

Standard error for all measured biological parameters is displayed in the appropriate figures. Student's t-test was utilized to determine the significance between groups. Statistical analysis was performed with Microsoft Excel. Significance was defined at the level of p<0.05.

## References

[1] Hemminki K, Lenner P, Sundquist J, Bermejo JL (2008) Risk of subsequent solid tumors after non-hodgkin's lymphoma: Effect of diagnostic age and time since diagnosis. J Clin Oncol 26 (11) 1850–18577. 18347006 10.1200/JCO.2007.14.6068

[2] Ettinger DS, Akerley W, Borghaei H, Chang AC, Cheney RT, et al (2013) Non-small cell lung cancer, version 2. J Natl Compr Canc Netw 11 (6) 645. 10.6004/jnccn.2013.0084 23744864

[3] Huang C (ed) (2012) Protein phosphorylation in human Health.

[4] Haupt S, Berger M, Goldberg Z, Haupt Y (2003) Apoptosis - the p53 network. J Cell Sci 16: 4077–4085. 10.1242/jcs.00739 12972501

[5] Schuler M, Green DR (2001) Mechanisms of p53-dependent apoptosis. Biochem Soc Trans 29: 684–688. 11709054 10.1042/0300-5127:0290684

[6] Shen Y, White E (2001) P53-dependent apoptosis pathways. Adv Cancer Res 82: 55–84. 11447765 10.1016/s0065-230x(01)82002-9

[7] Benchimol S (2001) p53-dependent pathways of apoptosis. Cell Death Differ 8 (11) 1049–1051. 11687883 10.1038/sj.cdd.4400918

[8] Brachman DG, Hallahan DE, Beckett MA, Yandell DW, Weichselbaum RR (1991) p53 gene mutations and abnormal retinoblastoma protein in radiation-induced human sarcomas. Cancer Res 51 (23 Pt 1) 6393–6396. 1933904

[9] Brash DE, Rudolph JA, Simon JA, Lin A, McKenna GJ, et al (1991) A role for sunlight in skin cancer: UV-induced p53 mutations in squamous cell carcinoma. Proc Natl Acad Sci U S A 88 (22) 10124–10128. 1946433 10.1073/pnas.88.22.10124 PMC52880

[10] Chiba I, Takahashi T, Nau MM, D'Amico D, Curiel DT, et al (1990) Mutations in the p53 gene are frequent in primary, resected non-small cell lung cancer. Lung cancer study group. Oncogene 5 (10) 1603–1610. 1979160

[11] DeLeo AB, Jay G, Appella E, Dubois GC, Law LW, et al (1979) Detection of a transformation-related antigen in chemically induced sarcomas and other transformed cells of the mouse. Proc Natl Acad Sci U S A 76 (5) 2420–2424. 221923 10.1073/pnas.76.5.2420 PMC383613

[12] Elbendary AA, Cirisano FD, Evans AC Jr, Davis PL, Iglehart JD, et al (1996) Relationship between p21 expression and mutation of the p53 tumor suppressor gene in normal and malignant ovarian epithelial cells. Clin Cancer Res 2 (9) 1571–1575. 9816335

[13] Fearon ER, Hamilton SR, Vogelstein B (1987) Clonal analysis of human colorectal tumors. Science 238 (4824) 193–197. 2889267 10.1126/science.2889267

[14] Gao WM, Mady HH, Yu GY, Siegfried JM, Luketich JD, et al (2003) Comparison of p53 mutations between adenocarcinoma and squamous cell carcinoma of the lung: Unique spectra involving G to A transitions and G to T transversions in both histologic types. Lung Cancer 40 (2) 141–150. 12711114 10.1016/s0169-5002(03)00035-7

[15] Gasco M, Shami S, Crook T (2002) The p53 pathway in breast cancer. Breast Cancer Res 4 (2) 70–76. 11879567 10.1186/bcr426 PMC138723

[16] Giglia-Mari G, Sarasin A (2003) TP53 mutations in human skin cancers. Hum Mutat 21 (3) 217–228. 12619107 10.1002/humu.10179

[17] Greenblatt MS, Bennett WP, Hollstein M, Harris CC (1994) Mutations in the p53 tumor suppressor gene: Clues to cancer etiology and molecular pathogenesis. Cancer Res 54 (18) 4855–4878. 8069852

[18] Hainaut P, Hollstein M (2000) p53 and human cancer: The first ten thousand mutations. Adv Cancer Res 77: 81–137. 10549356 10.1016/s0065-230x(08)60785-x

[19] Hollstein M, Sidransky D, Vogelstein B, Harris CC (1993) p53 mutations in human cancers. Science 253 (5015) 49–53. 10.1126/science.1905840 1905840

[20] Iggo R, Gatter K, Bartek J, Lane D, Harris AL (1990) Increased expression of mutant forms of p53 oncogene in primary lung cancer. Lancet 335 (8691) 675–679. 1969059 10.1016/0140-6736(90)90801-b

[21] Kozaki K, Miyaishi O, Tsukamoto T, Tatematsu Y, Hida T, et al (2000) Establishment and characterization of a human lung cancer cell line NCI-H460-LNM35 with consistent lymphogenous metastasis via both subcutaneous and orthotopic propagation. Cancer Res 60 (9) 2535–2540. 10811136

[22] Lehmann BD, Pietenpol JA (2012) Targeting mutant p53 in human tumors. J Clin Oncol 30 (29) 3648–3650. 22965952 10.1200/JCO.2012.44.0412

[23] Levine AJ, Vosburgh E (2008) P53 mutations in lymphomas: Position matters. Blood 112 (8) 2997–2998. 18840714 10.1182/blood-2008-07-167718

[24] Nakazawa H, English D, Randell PL, Nakazawa K, Martel N, et al (1994) UV and skin cancer: Specific p53 gene mutation in normal skin as a biologically relevant exposure measurement. Proc Natl Acad Sci U S A 91 (1) 360–364. 8278394 10.1073/pnas.91.1.360 PMC42947

[25] Richiardi L, Scelo G, Boffetta P, Hemminki K, Pukkala E, et al (2007) Second malignancies among survivors of germ-cell testicular cancer: A pooled analysis between 13 cancer registries. Int J Cancer 120 (3) 623–631. 17096341 10.1002/ijc.22345

[26] Rodrigues NR, Rowan A, Smith ME, Kerr IB, Bodmer WF, et al (1990) P53 mutations in colorectal cancer. Proc Natl Acad Sci U S A 87 (19) 7555–7559. 1699228 10.1073/pnas.87.19.7555 PMC54786

[27] Shi Y, Fu X, Hua Y, Han Y, Lu Y, et al (2012) The side population in human lung cancer cell line NCI-H460 is enriched in stem-like cancer cells. PLoS One 7 (3) e33358. 22428030 10.1371/journal.pone.0033358 PMC3302833

[28] Soussi T, Dehouche K, Beroud C (2000) P53 website and analysis of P53 gene mutations in human cancer: Forging a link between epidemiology and carcinogenesis. Hum Mutat 15 (1) 105–113. 10612830 10.1002/(SICI)1098-1004(200001)15:1<105::AID-HUMU19>3.0.CO;2-G

[29] Takahashi T, Nau MM, Chiba I, Birrer MJ, Rosenberg RK, et al (1989) P53: A frequent target for genetic abnormalities in lung cancer. Science 246 (4929) 491–494. 2554494 10.1126/science.2554494

[30] Vogelstein B, Fearon ER, Hamilton SR, Kern SE, Preisinger AC, et al (1988) Genetic alterations during colorectal-tumor development. N Engl J Med 319 (9) 525–532. 2841597 10.1056/NEJM198809013190901

[31] Wang X, Christiani DC, Wiencke JK, Fischbein M, Xu X, et al (1995) Mutations in the p53 gene in lung cancer are associated with cigarette smoking and asbestos exposure. Cancer Epidemiol Biomarkers Prev 4 (5) 543–548. 7549812

[32] Whibley C, Pharoah PD, Hollstein M (2009) P53 polymorphisms: Cancer implications. Nat Rev Cancer 9 (2) 95–107. 19165225 10.1038/nrc2584

[33] Baker SJ, Fearon ER, Nigro JM, Hamilton SR, Preisinger AC, et al (1989) Chromosome 17 deletions and p53 gene mutations in colorectal carcinomas. Science 244 (4901) 217–221. 2649981 10.1126/science.2649981

[34] Armstrong MB, Bian X, Liu Y, Subramanian C, Ratanaproeksa AB, et al (2006) Signaling from p53 to NF-kappa B determines the chemotherapy responsiveness of neuroblastoma. Neoplasia 8 (11) 964–974. 17215959 PMC1764827

[35] Murphy SH, Suzuki K, Downes M, Welch GL, De Jesus P, et al (2011) Tumor suppressor protein (p)53, is a regulator of NF-kappaB repression by the glucocorticoid receptor. Proc Natl Acad Sci U S A 108 (41) 17117–17122. 21949408 10.1073/pnas.1114420108 PMC3193198

[36] Ryan KM, Ernst MK, Rice NR, Vousden KH (2000) Role of NF-kappaB in p53-mediated programmed cell death. Nature 404 (6780) 892–897. 10786798 10.1038/35009130

[37] Arizti P, Fang L, Park I, Yin Y, Solomon E, et al (2000) Tumor suppressor p53 is required to modulate BRCA1 expression. Mol Cell Biol 20 (20) 7450–7459. 11003642 10.1128/mcb.20.20.7450-7459.2000 PMC86298

[38] De Luca P, Moiola CP, Zalazar F, Gardner K, Vazquez ES, et al (2013) BRCA1 and p53 regulate critical prostate cancer pathways. Prostate Cancer Prostatic Dis 16 (3) 233–238. 23670255 10.1038/pcan.2013.12 PMC6944434

[39] Kumar P, Mukherjee M, Johnson JP, Patel M, Huey B, et al (2012) Cooperativity of rb, Brca1, and p53 in malignant breast cancer evolution. PLoS Genet 8 (11) e1003027. 23173005 10.1371/journal.pgen.1003027 PMC3500050

[40] Xu X, Qiao W, Linke SP, Cao L, Li WM, et al (2001) Genetic interactions between tumor suppressors Brca1 and p53 in apoptosis, cell cycle and tumorigenesis. Nat Genet 28 (3) 266–271. 11431698 10.1038/90108

[41] Zhang H, Somasundaram K, Peng Y, Tian H, Zhang H, et al (1991) BRCA1 physically associates with p53 and stimulates its transcriptional activity. Oncogene 16 (13) 1713–1721. 10.1038/sj.onc.1201932 9582019

[42] Nagai W, Okita N, Matsumoto H, Okado H, Oku M, et al (2012) Reversible induction of PARP1 degradation by p53-inducible cis-imidazoline compounds. Biochem Biophys Res Commun 421 (1) 15–19. 22465010 10.1016/j.bbrc.2012.03.091

[43] Valenzuela MT, Guerrero R, Nunez MI, Ruiz De Almodovar JM, Sarker M, et al (2002) PARP-1 modifies the effectiveness of p53-mediated DNA damage response. Oncogene 21 (7) 1108–1116. 11850828 10.1038/sj.onc.1205169

[44] Kubbutat MH, Ludwig RL, Ashcroft M, Vousden KH (1998) Regulation of Mdm2-directed degradation by the C terminus of p53. Mol Cell Biol 18 (10) 5690–5698. 9742086 10.1128/mcb.18.10.5690 PMC109155

[45] Maki CG (1999) Oligomerization is required for p53 to be efficiently ubiquitinated by MDM2. J Biol Chem 274 (23) 16531–16535. 10347217 10.1074/jbc.274.23.16531

[46] Aylon Y, Oren M (2011) New plays in the p53 theater. Curr Opin Genet Dev 21 (1) 86–92. 21317061 10.1016/j.gde.2010.10.002 PMC3059112

[47] Rivlin N, Brosh R, Oren M, Rotter V (2011) Mutations in the p53 tumor suppressor gene: Important milestones at the various steps of tumorigenesis. Genes Cancer 2 (4) 466–474. 21779514 10.1177/1947601911408889 PMC3135636

[48] Huang S, Benavente S, Armstrong EA, Li C, Wheeler DL, et al (2011) p53 modulates acquired resistance to EGFR inhibitors and radiation. Cancer Res 71 (22) 7071–7079. 22068033 10.1158/0008-5472.CAN-11-0128 PMC3229180

[49] Berkson RG, Hollick JJ, Westwood NJ, Woods JA, Lane DP, et al (2005) Pilot screening programme for small molecule activators of p53. Int J Cancer 115 (5) 701–710. 15729694 10.1002/ijc.20968

[50] Petitjean A, Mathe E, Kato S, Ishioka C, Tavtigian SV, et al (2007) Impact of mutant p53 functional properties on TP53 mutation patterns and tumor phenotype: Lessons from recent developments in the IARC TP53 database. Hum Mutat 28 (6) 622–629. 17311302 10.1002/humu.20495

[51] Levine AJ, Wu MC, Chang A, Silver A, Attiyeh EF, et al (1995) The spectrum of mutations at the p53 locus. Evidence for tissue-specific mutagenesis, selection of mutant alleles, and a “gain of function” phenotype. Ann N Y Acad Sci 768: 111–128. 8526340 10.1111/j.1749-6632.1995.tb12115.x

[52] Chene P (2001) The role of tetramerization in p53 function. Oncogene 20 (21) 2611–2617. 11420672 10.1038/sj.onc.1204373

[53] Shieh SY, Ahn J, Tamai K, Taya Y, Prives C (2000) The human homologs of checkpoint kinases Chk1 and Cds1 (Chk2) phosphorylate p53 at multiple DNA damage-inducible sites. Genes Dev 14 (3) 289–300. 10673501 PMC316358

[54] Chene P (2000) Fast, qualitative analysis of p53 phosphorylation by protein kinases. BioTechniques 28 (2) 240–242. 10683732

[55] Maki CG (1999) Oligomerization is required for p53 to be efficiently ubiquitinated by MDM2. J Biol Chem 274 (23) 16531–16535. 10347217 10.1074/jbc.274.23.16531

[56] Balagurumoorthy P, Sakamoto H, Lewis MS, Zambrano N, Clore GM, et al (1995) Four p53 DNA-binding domain peptides bind natural p53-response elements and bend the DNA. Proc Natl Acad Sci U S A 2 (19) 8591–8595. 10.1073/pnas.92.19.8591 PMC41012 7567980

[57] Bargonetti J, Manfredi JJ, Chen X, Marshak DR, Prives C (1993) A proteolytic fragment from the central region of p53 has marked sequence-specific DNA-binding activity when generated from wild-type but not from oncogenic mutant p53 protein. Genes Dev 7 (12B) 2565–2574. 8276239 10.1101/gad.7.12b.2565

[58] McLure KG, Lee PW (1998) How p53 binds DNA as a tetramer. EMBO J 17 (12) 3342. 9628871 10.1093/emboj/17.12.3342 PMC1170672

[59] Pavletich NP, Chambers KA, Pabo CO (1993) The DNA-binding domain of p53 contains the four conserved regions and the major mutation hot spots. Genes Dev 7 (12B) 2556–2564. 8276238 10.1101/gad.7.12b.2556

[60] Sang BC, Chen JY, Minna J, Barbosa MS (1994) Distinct regions of p53 have a differential role in transcriptional activation and repression functions. Oncogene 9 (3) 853–859. 8108128

[61] Shaulian E, Zauberman A, Milner J, Davies EA, Oren M (1993) Tight DNA binding and oligomerization are dispensable for the ability of p53 to transactivate target genes and suppress transformation. EMBO J 12 (7) 2789–2797. 8334995 10.1002/j.1460-2075.1993.tb05940.x PMC413529

[62] Stenger JE, Tegtmeyer P, Mayr GA, Reed M, Wang Y, et al (1994) p53 oligomerization and DNA looping are linked with transcriptional activation. EMBO J 13 (24) 6011–6020. 7813439 10.1002/j.1460-2075.1994.tb06947.x PMC395578

[63] Shi Y, Fu X, Hua Y, Han Y, Lu Y, et al (2012) The side population in human lung cancer cell line NCI-H460 is enriched in stem-like cancer cells. PLoS One 7 (3) e33358. 22428030 10.1371/journal.pone.0033358 PMC3302833

[64] Bostrom PJ, Soloway MS (2007) Secondary cancer after radiotherapy for prostate cancer: Should we be more aware of the risk? Eur Urol 52 (4) 973–982. 17644245 10.1016/j.eururo.2007.07.002

[65] Brown LM, Chen BE, Pfeiffer RM, Schairer C, Hall P, et al (2007) Risk of second non-hematological malignancies among 376,825 breast cancer survivors. Breast Cancer Res Treat 106 (3) 439–451. 17277968 10.1007/s10549-007-9509-8

[66] Chaturvedi AK, Engels EA, Gilbert ES, Chen BE, Storm H, et al (2007) Second cancers among 104,760 survivors of cervical cancer: Evaluation of long-term risk. J Natl Cancer Inst 99 (21) 1634–1643. 17971527 10.1093/jnci/djm201

[67] De Benedetti VM, Travis LB, Welsh JA, van Leeuwen FE, Stovall M, et al (1996) p53 mutations in lung cancer following radiation therapy for Hodgkin's disease. Cancer Epidemiol Biomarkers Prev 5 (2) 93–98. 8850268

[68] Fogelfeld L, Bauer TK, Schneider AB, Swartz JE, Zitman R (1996) P53 gene mutations in radiation-induced thyroid cancer. J Clin Endocrinol Metab 81 (8) 3039–3044. 8768871 10.1210/jcem.81.8.8768871

[69] Hall EJ, Wuu CS (2003) Radiation-induced second cancers: The impact of 3D-CRT and IMRT. Int J Radiat Oncol Biol Phys 56 (1) 83–88. 12694826 10.1016/s0360-3016(03)00073-7

[70] Mudie NY, Swerdlow AJ, Higgins CD, Smith P, Qiao Z, et al (2006) Risk of second malignancy after non-hodgkin's lymphoma: A british cohort study. J Clin Oncol 24 (10) 1568–1574. 16520465 10.1200/JCO.2005.04.2200

[71] Nieder AM, Porter MP, Soloway MS (2008) Radiation therapy for prostate cancer increases subsequent risk of bladder and rectal cancer: A population based cohort study. J Urol 180 (5) 2005. 18801517 10.1016/j.juro.2008.07.038

[72] Pinto EM, Siqueira SA, Cukier P, Fragoso MC, Lin CJ, et al (2011) Possible role of a radiation-induced p53 mutation in a nelson's syndrome patient with a fatal outcome. Pituitary 14 (4) 400–404. 19655257 10.1007/s11102-009-0194-y

[73] Travis LB, Fossa SD, Schonfeld SJ, McMaster ML, Lynch CF, et al (2005) Second cancers among 40,576 testicular cancer patients: Focus on long-term survivors. J Natl Cancer Inst 97 (18) 1354–1365. 16174857 10.1093/jnci/dji278

[74] Tward JD, Wendland MM, Shrieve DC, Szabo A, Gaffney DK (2006) The risk of secondary malignancies over 30 years after the treatment of non-hodgkin lymphoma. Cancer 107 (1) 108–115. 16708354 10.1002/cncr.21971

[75] Jung IL, Kang HJ, Kim KC, Kim IG (2010) PTEN/pAkt/p53 signaling pathway correlates with the radioresponse of non-small cell lung cancer. Int J Mol Med 25 (4) 517–523. 20198299 10.3892/ijmm_00000372

[76] Lee YS, Oh JH, Yoon S, Kwon MS, Song CW, et al (2010) Differential gene expression profiles of radioresistant non-small-cell lung cancer cell lines established by fractionated irradiation: Tumor protein p53-inducible protein 3 confers sensitivity to ionizing radiation. Int J Radiat Oncol Biol Phys 77 (3) 858–866. 20510196 10.1016/j.ijrobp.2009.12.076

[77] van Baardwijk A, Bosmans G, Bentzen SM, Boersma L, Dekker A, et al (2008) Radiation dose prescription for non-small-cell lung cancer according to normal tissue dose constraints: An in silico clinical trial. Int J Radiat Oncol Biol Phys 71 (4) 1103–1110. 18258382 10.1016/j.ijrobp.2007.11.028

[78] Lubin DJ, Butler JS, Loh SN (2010) Folding of tetrameric p53: Oligomerization and tumorigenic mutations induce misfolding and loss of function. J Mol Biol 395 (4) 705–716. 19913028 10.1016/j.jmb.2009.11.013

[79] Oren M, Rotter V (2010) Mutant p53 gain-of-function in cancer. Cold Spring Harb Perspect Biol 2 (2) a001107. 20182618 10.1101/cshperspect.a001107 PMC2828285

[80] Macleod KF, Sherry N, Hannon G, Beach D, Tokino T, et al (1995) p53-dependent and independent expression of p21 during cell growth, differentiation, and DNA damage. Genes Dev 9 (8) 935–944. 7774811 10.1101/gad.9.8.935

[81] Blagosklonny MV (2000) P53 from complexity to simplicity: Mutant P53 stabilization, gain-of-function, and dominant-negative effect. FASEB J 14 (13) 1901–1907. 11023974 10.1096/fj.99-1078rev

[82] Brachmann RK, Vidal M, Boeke JD (1996) Dominant-negative p53 mutations selected in yeast hit cancer hot spots. Proc Natl Acad Sci U S A 93 (9) 4091–4095. 8633021 10.1073/pnas.93.9.4091 PMC39492

[83] de Vries A, Flores ER, Miranda B, Hsieh HM, van Oostrom CT, et al (2002) Targeted point mutations of p53 lead to dominant-negative inhibition of wild-type p53 function. Proc Natl Acad Sci U S A 99 (5) 2948–2953. 11867759 10.1073/pnas.052713099 PMC122453

[84] Willis A, Jung EJ, Wakefield T, Chen X (2004) Mutant p53 exerts a dominant negative effect by preventing wild-type p53 from binding to the promoter of its target genes. Oncogene 23 (13) 2330–2338. 14743206 10.1038/sj.onc.1207396

[85] Shaulian E, Zauberman A, Ginsberg D, Oren M (1992) Identification of a minimal transforming domain of p53: Negative dominance through abrogation of sequence-specific DNA binding. Mol Cell Biol 12 (12) 5581–5592. 1448088 10.1128/mcb.12.12.5581-5592.1992 PMC360497

[86] Chene P (1998) In vitro analysis of the dominant negative effect of p53 mutants. J Mol Biol 281 (2) 205–209. 9698540 10.1006/jmbi.1998.1897

[87] Milner J, Medcalf EA (1991) Cotranslation of activated mutant p53 with wild type drives the wild-type p53 protein into the mutant conformation. Cell 65 (5) 765–774. 2040013 10.1016/0092-8674(91)90384-b

[88] Chene P, Bechter E (1999) P53 mutants without a functional tetramerisation domain are not oncogenic. J Mol Biol 286 (5) 1269–1274. 10064694 10.1006/jmbi.1999.2563

[89] Chene P, Mittl P, Grutter M (1997) In vitro structure-function analysis of the beta-strand 326–333 of human p53. J Mol Biol 273 (4) 873–881. 9367778 10.1006/jmbi.1997.1360

[90] Ko JL, Cheng YW, Chang SL, Su JM, Chen CY, et al (2000) MDM2 mRNA expression is a favorable prognostic factor in non-small-cell lung cancer. Int J Cancer 89 (3) 265–270. 10861503 10.1002/1097-0215(20000520)89:3<265::aid-ijc9>3.0.co;2-n

